# BCG‐induced cytokine release in bladder cancer cells is regulated by Ca^2+^ signaling

**DOI:** 10.1002/1878-0261.12397

**Published:** 2018-12-13

**Authors:** Cristián Ibarra, Marie Karlsson, Simone Codeluppi, Manuel Varas‐Godoy, Songbai Zhang, Lauri Louhivuori, Sara Mangsbo, Arad Hosseini, Navid Soltani, Rahim Kaba, T. Kalle Lundgren, Abolfazl Hosseini, Nobuyuki Tanaka, Mototsugu Oya, Peter Wiklund, Ayako Miyakawa, Per Uhlén

**Affiliations:** ^1^ Department of Medical Biochemistry and Biophysics Karolinska Institutet Stockholm Sweden; ^2^ Department of Physiology and Pharmacology Karolinska Institutet Stockholm Sweden; ^3^ Centro de Investigacion Biomedica Faculty of Medicine Universidad de los Andes Santiago Chile; ^4^ Department of Pharmaceutical Biosciences Science for Life Laboratory Uppsala University Sweden; ^5^ Department of Molecular Medicine and Surgery Karolinska University Hospital Stockholm Sweden; ^6^ Department of Urology Keio University School of Medicine Tokyo Japan; ^7^ Keio University Graduate School of Medicine Tokyo Japan

**Keywords:** BCG, calcium signaling, TLR4, urinary bladder cancer

## Abstract

Bacillus Calmette–Guérin (BCG) is widely used in the clinic to effectively treat superficial urinary bladder cancer. However, a significant proportion of patients who fail to respond to BCG risk cystectomy or death. Though more than 3 million cancer treatments with BCG occur annually, surprisingly little is known about the initial signaling cascades activated by BCG. Here, we report that BCG induces a rapid intracellular Ca^2+^ (calcium ion) signal in bladder cancer cells that is essential for activating the transcription factor nuclear factor kappa‐light‐chain‐enhancer of activated B cells (NF‐κB) and for synthesizing and secreting proinflammatory cytokines, including interleukin 8 (IL‐8). A similar Ca^2+^ response was observed when cells were exposed to the supernatant of BCG. Studying cellular molecular mechanisms involved in the BCG signaling event, we found pivotal roles for phospholipase C and the Toll‐like receptor 4. Further assessment revealed that this signaling pathway induces synthesis of IL‐8, whereas exocytosis appeared to be controlled by global Ca^2+^ signaling. These results shed new light on the molecular mechanisms underlying BCG treatment of bladder cancer, which can help in improving therapeutic efficacy and reducing adverse side effects.

**Abbreviations**
bBCGsn: boiled BCGsn
BCG: Bacillus Calmette–Guérin
BCGsn: Supernatant of BCG
Ca^2+^: calcium ion
ER: endoplasmic reticulum
fBCGsn: frozen BCGsn
GPCR: G protein‐coupled receptor
IL: interleukin
InsP_3_: inositol 1,4,5‐trisphosphate
InsP_3_R: inositol 1,4,5‐trisphosphate receptor
MSD: Meso Scale Discovery
NF‐κB: nuclear factor kappa‐light‐chain‐enhancer of activated B cells
PI3K: phosphatidylinositol 3‐kinase
PLC: phospholipase C
ppBCGsn: protein‐precipitated BCGsn
qRT‐PCR: quantitative RT‐PCR
TLR4: toll‐like receptor 4

## 1 Introduction

Bacillus Calmette–Guérin (BCG), an attenuated strain of *Mycobacterium bovis*, is arguably the most effective therapy for high‐risk non‐muscle‐invasive urinary bladder cancer, although it was originally developed with the intention to produce a vaccine against tuberculosis (Kassouf and Kamat, 2004; Liu *et al*., 2009; Morales *et al*., 1976). Bladder cancer is the fourth most commonly diagnosed cancer in men and tenth most common cancer in women in the United States (Siegel *et al*., 2017). Approximately 75% of bladder cancers do not invade the smooth muscle (Kaufman *et al*., 2009; Simons *et al*., 2008) and are considered non‐muscle‐invasive bladder cancers. The standard treatment for non‐muscle‐invasive bladder cancer is transurethral resection of the bladder tumor, followed by intravesical therapy with BCG (Chang *et al*., 2016). However, a significant proportion of patients fail to respond to BCG therapy; their tumors are refractory or relapse and may become invasive or metastatic (Kaufman *et al*., 2009).

Since the first report of intravesical use of BCG, there have been strong efforts to understand the pharmacology and toxicology of this treatment, which have hitherto been elusive. BCG is a mixture containing viable microorganisms, bacterial fragments, intracellular content, and soluble secreted compounds. It is currently accepted that the antitumor activity of BCG is derived by a local non‐specific immunological boost that recruits immunocompetent cells (Redelman‐Sidi *et al*., 2014). Several studies report that BCG instillation results in a significant cell‐mediated response in the bladder, characterized by secretion of cytokines and recruitment of immune cells (Redelman‐Sidi *et al*., 2014). However, the exact sequence of events from the BCG instillation to tumor eradication has been only partially addressed, which has limited the development of new BCG derivatives with enhanced clinical efficacy and fewer adverse side effects.

Calcium (Ca^2+^) signaling has been reported to regulate cytokine release and tumor growth (Berridge *et al*., 1998; Monteith *et al*., 2017; Roderick and Cook, 2008; Uhlen *et al*., 2000). Through the concert of actions between Ca^2+^ channels and pumps, intracellular signals can be shaped in infinite ways (Uhlen and Fritz, 2010). Release of Ca^2+^ from intracellular endoplasmic reticulum (ER) Ca^2+^ stores mainly occurs through inositol 1,4,5‐trisphosphate (InsP_3_) receptors (InsP_3_Rs), which are activated when phosphatidylinositol 4,5‐bisphosphate is cleaved by phospholipase C (PLC) into InsP_3_ and diacylglycerol. The subsequent elevation of the cytosolic Ca^2+^ concentration can activate downstream effectors that control numerous biological processes in cells (Mengel *et al*., 2010).

## 2 Methods

### 2.1 Cell cultures

Human bladder tumors were collected from patients undergoing transurethral resection in the Urology Unit of Karolinska University Hospital. Primary cell cultures were prepared as previously described (Rahman *et al*., 1987). Briefly, tissue was finely minced, enzymatically digested, pipetted to disperse clumps, washed in phosphate‐buffered saline, and cultured in a specialized medium. All experiments were ethically approved (Dnr 2011/421‐31/1), and written consent was obtained from all participants. The study methodologies conformed to the standards set by the Declaration of Helsinki.

The urinary bladder cancer cell lines T24 (human), RT4 (human), and MB49 (mouse) were purchased from ATCC (American Type Culture Collection, Manassas, VA, USA) and were occasionally tested for mycoplasma (last tested in 2015). Cells were propagated according to their instructions, and all experiments were performed using cells between passage numbers 3 and 20.

### 2.2 BCG treatment

Bacillus Calmette–Guérin (2 × 10^8^–3 × 10^9^ cfu, Medac, Chicago, IL, USA) RIVM‐derived strain (1173‐P2) was resuspended in 5 mL of the cell line‐specific culture medium. The BCG mixture was then diluted 10 times to the final concentration of 4 × 10^6^–6 × 10^7^ cfu·mL^−1^ and treated to the cells for the indicated time period. To fractionate the BCG, it was centrifuged at 3000 *g* for 30 min to separate a BCG pellet and BCG supernatant (BCGsn). The BCGsn was frozen (fBCGsn) at ‐20 °C overnight, boiled (bBCGsn) at 100 °C for 1 h, or treated with acetone to precipitate protein (ppBCGsn). Proteins within ppBCGsn were separated into mass fractions: > 100, > 50, > 30, > 10, and > 3 kDa (Amicon tubes, Millipore, St Charles, MO, USA).

### 2.3 Reagents

The following reagents were used: cyclopiazonic acid (CPA, 50 μm, Tocris Bioscience, Bristol, UK); 2‐aminoethoxydiphenylborane (2APB, 100 μm, Tocris); U73122 (2–4 μm, Tocris, Abingdon, UK); U73343 (2–4 μm, Tocris); Edelfosine (ET‐18‐OCH3, 2–4 μm, Tocris); Wortmannin (1 μm, Tocris); Pertussis toxin (PTX, 1 μg·mL^−1^, Tocris); and 6‐(phenylsulfinyl)tetrazolo[1,5‐b]pyridazine (Ro 106‐9920, 0.01–100 μm, Tocris), Xestospongin D (Xesto, 5 μm, Tocris).

### 2.4 Calcium imaging

Cells were loaded with the Ca^2+^‐sensitive fluorescence indicator Fluo‐3/AM (5 μm, Invitrogen, Carlsbad, CA, USA) in Krebs‐Ringer solution (119 mm NaCl, 2.5 mm KCl, 2.5 mm CaCl_2_, 1.3 mm MgCl_2_, 1.0 mm NaH_2_PO_4_, 20.0 mm HEPES (pH 7.4), and 11.0 mm dextrose) at 37 °C with 5% CO_2_ for 30 min prior to experiments. The Ca^2+^ imaging was conducted at 37 °C in a heat‐controlled chamber (QE‐1, Warner Instruments, New Haven, CT, USA) with a confocal microscope Zeiss LSM510NLO META (Carl Zeiss, Jena, Germany) equipped with a 20x/0.8NA dipping lens (Carl Zeiss). Excitation was set at 488 nm and emission was detected at 510 nm. The sampling frequency was set to 0.1 Hz. carl zeiss software (Carl Zeiss) was used to analyze the acquired images. Experiments were performed in a Krebs‐Ringer buffer and all drugs were bath‐applied.

### 2.5 Cytokine measurements

The level of secreted cytokines in response to BCG was measured with a Meso Scale Discovery (MSD) MULTI‐SPOT Assay System (Meso Scale Discovery, Rockville, MD, USA). Cytokines were analyzed in the supernatants of human primary bladder cancer cultures by the Human ProInflammatory 9‐Plex Ultra‐Sensitive Kit and in mouse MB49 cells by the Mouse ProInflammatory 9‐Plex Ultra‐Sensitive Kit, following instructions from the manufacturer. Controls for standard curves were included with each plate. Data are presented as the means ± standard error of the mean of a minimum of four experiments.

### 2.6 Small interfering RNA

siRNA against PLCβ3 and PLCγ (Dharmacon, Lafayette, CO, USA) were used to knock down proteins. T24 cells were seeded in 25‐mm plates to 60% confluency, and siRNA (100 μm) was transfected into cells with Lipofectamine 2000 (Invitrogen) in Opti‐MEM (Invitrogen), according to the manufacturers’ instructions. The knockdown efficiency of siRNA was confirmed by western blotting (Fig. S1A,B).

### 2.7 Western blotting

Cells were lysed in a modified RIPA buffer for 20 min at 4 °C, and equal amounts of protein were separated on a 10% sodium dodecyl sulfate gel electrophoresis, followed by transfer to a PVDF membrane. The membranes were blocked in 5% milk or bovine serum albumin in TRIS‐buffered saline solution with 0.5% Tween‐20 for 1 h before incubation with primary antibodies (PLCβ3, 1 : 1000; PLCγ, 1 : 1000; β‐actin, 1 : 1000, Abcam, Cambridge, UK) overnight at 4 °C and further incubation with secondary antibodies (1 : 5000) for 1 h. Bands were detected with a chemiluminescence kit (Amersham Biosciences, GE Healthcare UK Limited, Bucks, UK) and an imaging system (Bio‐Rad, Hercules, CA, USA).

### 2.8 Luciferase reporter assays

The activity of nuclear factor kappa‐light‐chain‐enhancer of activated B cells (NF‐κB) was determined with a NF‐κB‐firefly‐luciferase reporter and CMV‐Renilla‐luciferase reporter constructs (Promega, Madison, WI, USA). MB49 cells were seeded at 1–2 × 10^4^ cells per well in 96‐well culture plates. On the following day, cells were co‐transfected with a firefly‐luciferase reporter construct with an interleukin 8 (IL‐8) promoter (or five copies of NF‐κB response element; 0.1 μg·well^−1^) and pCMV‐Renilla‐luciferase (0.005 μg·well^−1^) using Lipofectamine and PLUS reagents, according to the manufacturer's protocol (Invitrogen). Three hours after transfection, the medium was removed and replaced with complete Dulbecco's modified Eagle's medium for 24 h. The cells were preincubated with inhibitors for 24 h and then stimulated with BCG for 18 h. Then, cells were washed once with phosphate‐buffered saline and lysed in 25 μL Tropix lysis solution per well. Luciferase activity was determined with a Dual Luciferase Reporter Gene Assay (Promega) and a Wallac Victor^2^ 1420 Multilabel Counter according to the manufacturer's instructions (Wallac, Gaithersburg, MD, USA). Renilla‐luciferase activity was analyzed to verify the reproducibility between quadruplicate transfections in all experiments.

### 2.9 Lentiviral vector production and *in vitro* transduction

Short hairpin RNA (shRNA) targeting the mouse Toll‐like receptor 4 (TLR4) mRNA, and a non‐related sequence (Control) were cloned into the lentiviral vector pLL3.7‐mRuby (Rubinson *et al*., 2003). The shRNA sequences were sh1TLR4: 5′‐ GCATAGAGGTAGTTCCTAATA ‐3′, sh2TLR4: 5′‐CTTCACTACAGAGACTTTA‐3′, and shControl: 5′‐TTCTCCGAACGTGTCACGT‐3′. Lentivirus was produced by co‐transfecting the packaging vector pΔ8.91 and the envelope vector pCMV‐VSVg with the pLL3.7.mRuby2 into HEK293FT cells (Invitrogen). The resulting supernatant was harvested after 60 h, concentrated by ultracentrifugation (95 min at 103.864 ***g*** in a Beckman JS‐24.38 rotor) and resuspended in 100 μL of RPMI 1640 medium. MB49 cells were transduced and selected by cell sorting (BD FACS Aria II cell sorter) targeting the mRuby reporter. Downregulation of TLR4 was confirmed by quantitative RT‐PCR (qRT‐PCR; Fig. S1C). Briefly, total RNA from transduced MB49 cells was isolated with the RNeasy Mini Kit (QIAGEN, Hilden, Germany) and reverse transcribed with the Superscript II Kit (Invitrogen). qRT‐PCR assays were performed with the SYBR^®^ green PCR master mix (Applied Biosystems, Foster City, CA, USA) in an ABI PRISM 7900HT sequence detection system (Applied Biosystems). The expression of the target mRNA was normalized to glyceraldehyde 3‐phosphate dehydrogenase (GAPDH). The primers used were mouse TLR4 forward: 5′‐CACTGTTCTTCTCCTGCCTGAC‐3′, mouse TLR4 reverse: 5′‐CCTGGGGAAAAACTCTGGATAG‐3′, mouse GAPDH forward: 5′‐TGACCTCAACTACATGGTCTACA‐3′, and mouse GAPDH reverse: 5′‐ CTTCCCATTCTCGGCCTTG‐3′. sh2TLR4 was selected for further experiments.

### 2.10 Statistical analysis

The Ca^2+^ recording data were normalized and cells were considered responsive to a treatment if the mean fluorescence was increased by at least 50% over the baseline. All the data are presented as the means ± SEM. Unless otherwise stated, at least three biological repeats were performed for all of the cell culture experiment. Groups were compared by Student's two‐tailed unpaired *t*‐test or one‐way ANOVA, with a *P*‐value < 0.05 as the limit for statistical significance.

## 3 Results

### 3.1 BCG elevates the cytosolic Ca^2+^ concentration

**Figure 1:**
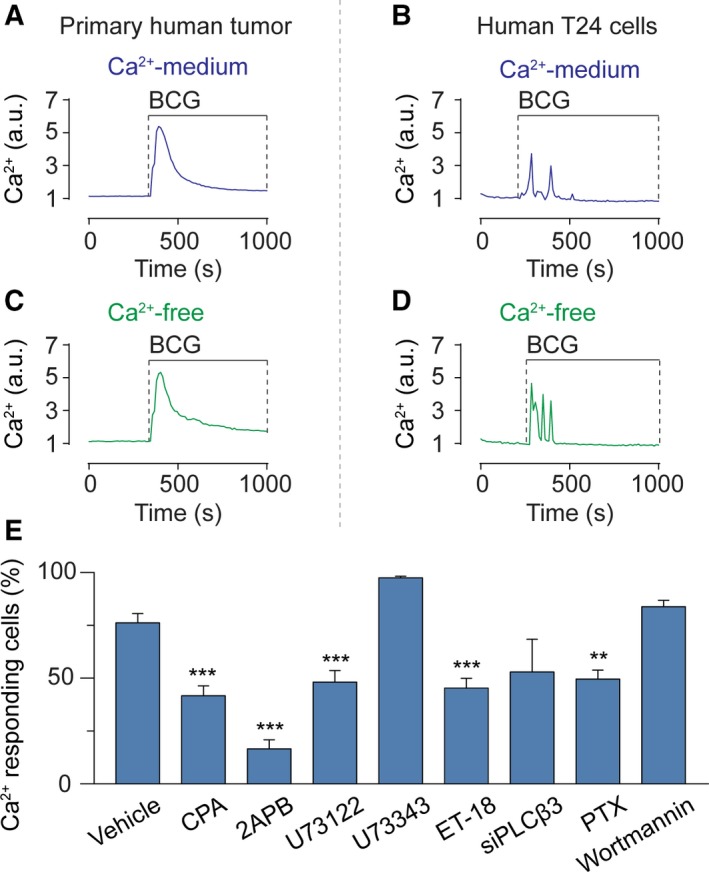
BCG evokes intracellular Ca^2+^ signaling in bladder cancer cells. Primary human bladder cancer cells (A) and human T24 cells (B) exposed to BCG (4 × 10^6^–6 × 10^7^ cfu·mL^−1^) in a Ca^2+^‐containing buffer exhibit Ca^2+^ signaling. Primary human bladder cancer cells (C) and human T24 cells (D) exposed to BCG (4 × 10^6^–6 × 10^7^ cfu·mL^−1^) in a Ca^2+^‐free buffer also exhibit Ca^2+^ signaling. (E) The number of human T24 cells responding to BCG with Ca^2+^ signaling was significantly reduced by the inhibitors CPA, 2APB, U73122, ET‐18‐OCH3 (ET‐18), and PTX, whereas wortmannin failed to significantly reduce the number of active cells. Results are means ± SEM of measurements from at least three separate cell cultures. *P < 0.05, **P < 0.01, ***P < 0.001 (Student's *t*‐test).

To test the influence of BCG on Ca^2+^ homeostasis in urinary bladder cancer cells, we collected tumor samples from patients treated with transurethral resection. We prepared primary cultures of human bladder cancer cells and loaded them with Fluo‐3/AM to monitor the cytosolic Ca^2+^ concentration with time‐lapse fluorescence microscopy. First, the basal Ca^2+^ level was recorded for ~ 5 min, and then, a clinically relevant preparation of BCG (4 × 10^6^–6 × 10^7^ cfu·mL^−1^) was applied to the cells. A rapid cytosolic Ca^2+^ increase was observed when primary bladder cancer cells were exposed to BCG (Fig. 1A). Next, we assayed the Ca^2+^ response to BCG in T24 cells, a human cell line derived from poorly differentiated (grade III) bladder carcinoma (Bubenik *et al*., 1973), RT4 cells, a human cell line derived from a grade I urothelial carcinoma (Franks and Rigby, 1975), and MB49 cells, a murine model of bladder cancer (Summerhayes and Franks, 1979). BCG triggered Ca^2+^ responses in all these cell types (Figs 1B, S2A,B, and Movie S1). Interestingly, the Ca^2+^ responses induced by BCG showed oscillatory behaviors. To determine whether the rise in cytosolic Ca^2+^ was due to influx from the extracellular milieu, we repeated the experiment in a Ca^2+^‐free medium. Eliminating extracellular Ca^2+^ had no apparent effect on the BCG‐induced Ca^2+^ response (Fig. 1C,D), suggesting that the major Ca^2+^ source was intracellular stores.

The intracellular Ca^2+^ signaling pathway triggered by BCG was further scrutinized in human T24 cells, of which 76.2 ± 4.4% (*n *=* *2231, *N *=* *21) showed a Ca^2+^ response when exposed to BCG (Fig. 1E). First, internal ER Ca^2+^ stores were depleted by blocking the SERCA pump with CPA, which abolished the BCG‐induced Ca^2+^ signal (Figs 1E and S3A). Then, we inhibited InsP_3_Rs with 2APB or Xestospongin D, which abolished the response (Figs 1E and S3B,C). Additionally, blocking PLC with U73122 or ET‐18‐OCH_3_ completely eliminated the BCG‐induced response (Figs 1E and S3D). U73343, the inactive analog of U73122, had no effect (Figs 1E and S3E).

We next applied RNA silencing (siRNA) to pinpoint which isoform of PLC was involved in the signaling event. siRNA‐based knockdown of PLCβ3 inhibited the BCG‐induced Ca^2+^ response (Fig. S3F), whereas siRNA knockdown of PLCγ did not (Fig. S3G). Since G protein‐coupled receptors (GPCRs) and receptor tyrosine kinases interact with PLC, we then tested whether they played a role in BCG‐induced Ca^2+^ signaling by treating cells with PTX and the phosphatidylinositol 3‐kinase (PI3K) inhibitor wortmannin. PTX abolished the Ca^2+^ response (Figs 1E and S3H), while wortmannin did not (Figs 1E and S3I). The wortmannin experiment demonstrated that PI3K signaling was not required for the Ca^2+^ mobilization. Together, these data suggest that BCG triggers an intracellular Ca^2+^ response that is mediated by the release of Ca^2+^ from internal ER stores through a mechanism dependent on PLCβ3 and GPCRs.

### 3.2 BCG‐induced cytokine release is Ca^2+^ dependent

**Figure 2:**
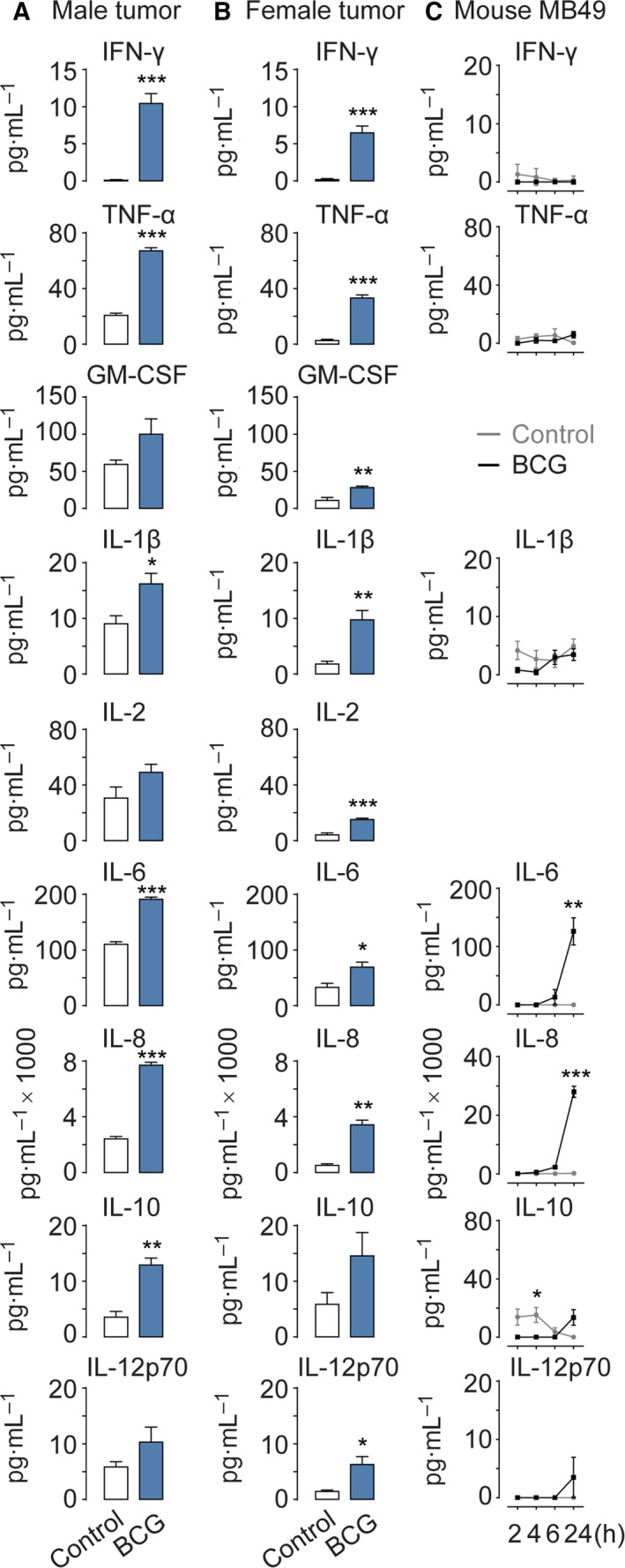
BCG triggers cytokine release in bladder cancer cells. Primary human bladder cancer cells derived from one male tumor (A) and one female tumor (B) or mouse MB49 cells (C) exposed to BCG (4 × 10^6^–6 × 10^7^ cfu·mL^−1^) secrete multiple cytokines, as compared to a control group. Results are means ± SEM of measurements from four separate cell cultures. *P < 0.05, **P < 0.01, ***P < 0.001 (Student's *t*‐test)

**Figure 3:**
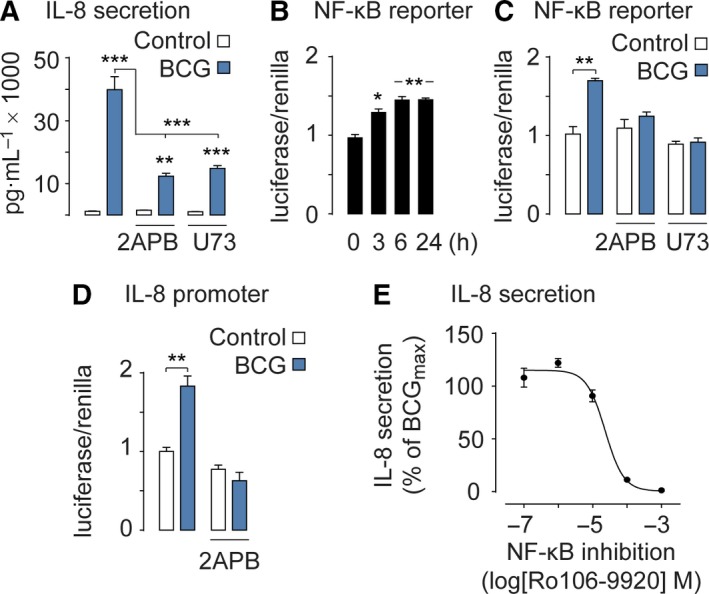
BCG activates NF‐κB and IL‐8. (A) BCG‐stimulated IL‐8 secretion is reduced when Ca^2+^ signaling is inhibited by 2APB or U73122 (U73). (B) NF‐κB reporter gene assay shows that NF‐κB is fully activated after 6 h of BCG treatment. (C) NF‐κB reporter gene assay shows that the NF‐κB activation is reduced when Ca^2+^ signaling is inhibited by 2APB or U73122 (U73). (D) BCG‐stimulated IL‐8 transcription is blocked when Ca^2+^ signaling is inhibited by 2APB. (E) BCG‐stimulated IL‐8 secretion levels are NF‐κB dependent as the inhibitor Ro106‐9920 hampers IL‐8 secretion in a dose‐dependent manner. Results are means ± SEM of measurements from at least three separate cell cultures. *P < 0.05, **P < 0.01, ***P < 0.001 (one‐way ANOVA)

We next sought out to test whether BCG‐induced cytokine release (Redelman‐Sidi *et al*., 2014) in bladder cancer cells was dependent on Ca^2+^ signaling. We decided to carry out these experiments on primary cultures of bladder cancer cells and MB49 cells, which is a widely used murine model of bladder cancer that is also sensitive to immunotherapy (Gunther *et al*., 1999; Kobayashi *et al*., 2015). Culturing primary human bladder cancer cells from a male tumor and a female tumor, we found that BCG induces release of a wide range of cytokines (Fig. 2A,B). In particular, IL‐6 and IL‐8 were released at significant levels in primary human bladder cancer cells as well as in mouse MB49 cells (Fig. 2C). BCG also induced a strong release of IFN‐γ, TNF‐α, IL‐1β, IL‐10, and IL‐12p70 in human bladder cancer cells. To determine whether the cytokine production was dependent on Ca^2+^ signaling, we measured IL‐8 release in MB49 cells pretreated with the inhibitors 2APB or U73122. Both drugs significantly hampered the BCG‐induced secretion of IL‐8 (Fig. 3A). Since NF‐κB is known to be regulated by Ca^2+^ signaling (Smedler and Uhlen, 2014) and to modulate IL‐8 (Karin *et al*., 2002), we then examined whether NF‐κB activation was induced by BCG. We determined that BCG induced a time‐dependent transcriptional activation of NF‐κB that leveled off after 6 h (Fig. 3B). This activation was indeed Ca^2+^ dependent, as 2APB and U73122 were able to hinder NF‐κB transcription induced by BCG (Fig. 3C).

Next, we analyzed the IL‐8 promoter activity. Inhibiting the intracellular Ca^2+^ release with 2APB abrogated IL‐8 transcription induced by BCG (Fig. 3D). To test whether the secretion of IL‐8 was dependent on NF‐κB activation, we gradually inhibited NF‐κB by varying the concentration of the NF‐κB inhibitor Ro106‐9920 together with a constant maximum dose of BCG. This experiment revealed a positive correlation between the NF‐κB activation and IL‐8 secretion (Fig. 3E).

These findings indicate that BCG‐evoked cytokine secretion is dependent on Ca^2+^ signaling and subsequent NF‐κB transcription.

### 3.3 The supernatant of BCG and TLR4 are activating IL‐8

**Figure 4:**
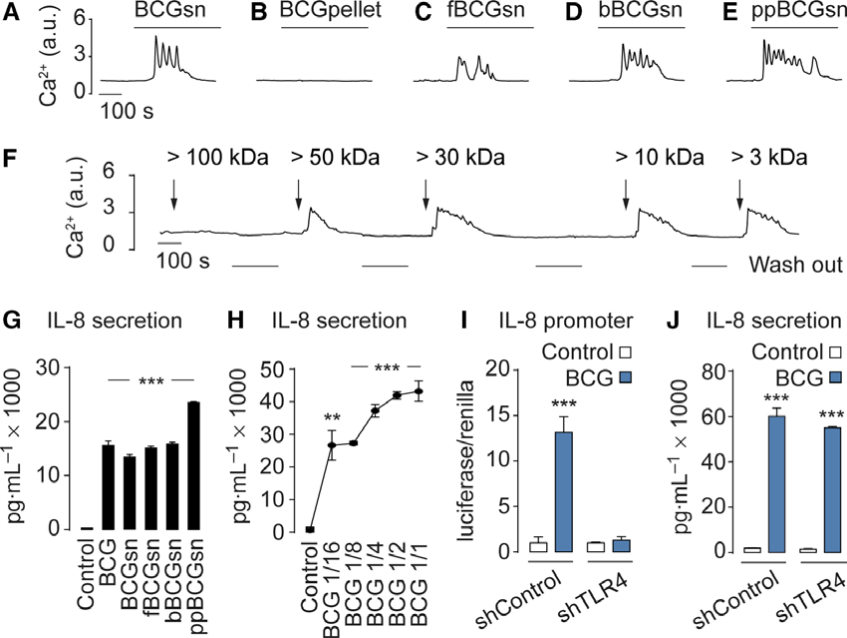
The BCGsn and TLR4 are key for BCG‐induced Ca^2+^ signaling. Assessment of Ca^2+^ signaling in mouse MB49 cells exposed to BCG preparations BCGsn (A), BCG pellet (B), fBCGsn (C), bBCGsn (D), ppBCGsn (E), or various weight fractions (F). (G) The BCG preparations that triggers Ca^2+^ signaling also stimulate IL‐8 secretion. (H) BCG at dilutions of 1/16, 1/8, 1/4, 1/2, and 1/1 activate IL‐8 in a dose‐dependent manner. TLR4 knockdown with shRNA (shTLR4) abolishes BCG‐induced IL‐8 transcription (I) but not IL‐8 secretion (J), compared to scramble shRNA controls (shControl). Results are means ± SEM of measurements from at least three separate cell cultures. *P < 0.05, **P < 0.01, ***P < 0.001 (Student's *t*‐test).

The BCG mixture used in the clinic contains viable microorganisms, bacterial fragments, intracellular content, and soluble secreted compounds. We decided to screen for key bioactive compounds within the BCG mixture responsible for triggering the Ca^2+^ signaling response. First, we prepared a BCGsn. To our surprise, exposing cells to the BCGsn resulted in a similar Ca^2+^ response to that observed with the entire BCG mixture (Fig. 4A). In contrast, exposing cells to the BCG pellet failed to elicit Ca^2+^ signaling (Fig. 4B). To test whether the response required enzymatic activity we prepared fBCGsn and to test whether it was protein dependent, we prepared bBCGsn and protein‐precipitated BCGsn (ppBCGsn). All three preparations were found to evoke Ca^2+^ responses in bladder cancer cells (Fig. 4C,E). Next, we fractionated ppBCGsn according to the following molecular weights: > 100, > 50, > 30, > 10, and > 3 kDa. All fractions except > 100 kDa triggered Ca^2+^ responses (Fig. 4F), suggesting that the bioactive compound(s) triggering the Ca^2+^ response was lighter than 100 kDa. Indeed, when performing a polyacrylamide gel electrophoresis of the different BCG fractions, a majority of the bands detected were lighter than 100 kDa (Fig. S4).

We then tested whether the various BCG fractions could also activate IL‐8. Treating cells with BCGsn, fBCGsn, bBCGsn, or ppBCGsn showed a similar IL‐8 response as treating with whole BCG (Fig. 4G). When exposing cells to a series of different BCG concentrations, we found that the IL‐8 secretion stimulated by BCG was dose dependent (Fig. 4H). Next, we investigated whether TLR4 was involved the release of IL‐8. We used lentivirus encoding shRNA against TLR4 (shTLR4), as well as scramble shRNA as a control (shControl). Infecting cells with shTLR4 and exposing them to BCG completely abolished the IL‐8 promoter activity (Fig. 4I). In contrast, measuring IL‐8 secretion under the same conditions demonstrated no effect of TLR4 knockdown (Fig. 4J). Together, these observations indicate that bioactive compounds within the BCGsn are evoking Ca^2+^ signaling and that TLR4 is controlling the synthesis, not the exocytosis, of IL‐8.

## 4 Discussion

Despite the frequent clinical usage of BCG to eradicate bladder cancer, little is known about its mechanism of action. Nevertheless, several studies report that BCG causes a local immune response reaction in the bladder (de Boer *et al*., 1997; Jackson *et al*., 1995; Redelman‐Sidi *et al*., 2014; Thalmann *et al*., 2000). BCG induces cancer cells in the bladder to secrete cytokines and chemokines, which attract cells of the immune system (Horinaga *et al*., 2005; Kamat *et al*., 2009; Liu *et al*., 2009). The cancer cells are subsequently killed by cytotoxic cells recruited to the site of the inflammatory response. IL‐6 and IL‐8 rapidly appear in the urine of BCG‐instilled patients and are believed to initiate the host immune response (Kamat *et al*., 2016). In this study, we shed new light on the signaling mechanisms involved in the eradication of bladder cancer following BCG therapy.

The BCG mixture injected into the bladder of cancer patients contains a myriad of compounds, including fragments of bacteria and even semi‐live bacteria. Early publications report that one immunostimulatory compound in BCG is non‐methylated CG‐rich DNA fragments that can induce a potent immune activation (Yamamoto *et al*., 1992a,b). However, most likely several compounds within the BCG mixture are involved in the overall response. Interestingly, our data show that the BCG supernatant sBCG can stimulate cytosolic Ca^2+^ signaling and cytokine release. We identified TLR4 as an essential receptor for BCG‐induced Ca^2+^ signaling. Previously, expression of both TLR4 and TLR9 has been reported in human bladder cancer cells (Olbert *et al*., 2015). Our data show that inhibiting Ca^2+^ signaling with 2APB or knocking down TLR4 with shRNA completely blocks IL‐8 promoter activity. Surprisingly, knocking down TLR4 had no effect on IL‐8 secretion, whereas inhibiting Ca^2+^ signaling partly reduced secretion. This indicates that global Ca^2+^ signaling regulates exocytosis. However, more information about the various compounds within BCG and their actions on cells is necessary to fully understand the overall cellular response. We speculate that bioactive compounds within the BCG mixture can be counterproductive. Identifying such compounds can help reduce adverse side effects of BCG therapy as well as decrease the risk of cancer relapse or progression to more invasive disease.

Our data show that pertussis toxin and PLC can inhibit BCG‐induced Ca^2+^ signaling. It has been demonstrated that mice with orthotopic bladder tumors that receive a recombinant BCG expressing pertussis toxin have less tumor growth than animals receiving ordinary BCG (Chade *et al*., 2008). Interestingly, a new TLR transduction mechanism that involves Ca^2+^ signaling was recently reported (Shintani *et al*., 2013, 2014). TLR9 stimulation reduced ER Ca^2+^‐ATPase activity, modulating Ca^2+^ handling between the ER and mitochondria, which resulted in decreased mitochondrial ATP levels and activation of protective cellular machineries. It is possible that when treating bladder cancer patients with BCG, parallel protective machineries are activated that could hinder the cancer eradication process and explain why some patients fail to respond to BCG. One such parallel process might be the secretion of IL‐8, which can act as a tumorigenic and pro‐angiogenic factor, driving tumor growth (Singh and Lokeshwar, 2011; Xie, 2001; Zhao *et al*., 2017).

A major challenge with the BCG therapy is its severe side effects, such as BCG sepsis, immunosuppression, hematuria, active urinary tract infection, and mild cystitis (Kresowik and Griffith, 2009). Another disadvantage with the therapy is that a significant proportion (~ 40%) are refractory cancers that fail to respond to the BCG therapy (O'Donnell and Boehle, 2006; Packiam *et al*., 2017). Why some patients fail to respond is largely unknown. Based on our results, we speculate that concerted actions of multiple signaling pathways define the efficiency of the BCG therapy. BCG triggers a long chain of events that critically regulates each other. Patients with a malfunction in this cascade of events may have refractory cancer. We here present a much more comprehensive picture of the initial signaling event triggered by BCG, containing multiple interlinked signaling pathways. At the molecular level, it appears that bioactive compounds present in the BCGsn interact with TLR4 to trigger a Ca^2+^ response, which subsequently activates NF‐κB.

## 5 Conclusion

Our work opens the door to more extensive studies of the mechanism of action of BCG therapy for bladder cancer. By better understanding the BCG‐mediated signaling cascades, we will be better able to design more efficient therapeutic strategies with less adverse side effects when treating patients with bladder cancer.

## Ethics approval and consent to participate

All experiments were ethically approved by the Karolinska University Hospital Ethical Committee (Dnr 2011/421‐31/1) and all patients were informed.

## Conflict of interest

The authors declare no conflict of interest.

## Authors’ contributions

CI, MK, SC, and MVG designed research, performed research, and analyzed data. SZ, LL, NS, RK, and TKL performed research. ArH and AbH collected the tumor samples and clinical information. AbH, PW, SM, and MO advised the design of research. CI, MVG, SC, SM, AM, and PU prepared the manuscript. AM and PU designed the project. All authors read and approved the final manuscript.

## Supporting information


**Fig. S1.** Validating knock‐down efficiency of siRNA and shRNA.
**Fig. S2.** BCG evokes intracellular Ca2 +  signaling in bladder cancer cells.
**Fig. S3.** Scrutinizing the BCG‐induced Ca2 +  signal cascade in bladder cancer cells.
**Fig. S4.** Screening substances in BCG. Polyacrylamide gel electrophoresis of the BCG mixture.

**Movie S1.** BCG‐induced Ca2 +  signaling in bladder cancer cells.
