# Supplementary material for: BCG‐induced cytokine release in bladder cancer cells is regulated by Ca2+ signaling
Source: Mol Oncol. 2018 Dec 13;13(2):202–11. doi: 10.1002/1878-0261.12397 (PMC6360358; doi:10.1002/1878-0261.12397)

Supplementary Information

Supplementary figure legends

Supplementary Figure 1. Validating knock-down efficiency of siRNA and shRNA. Western blot analysis of PLC isoforms in T24 cells transfected with siRNAs against PLCβ3 (**A**) or PLCγ (**B**). β-actin was used as control. (**C**) Real-time PCR analysis of TLR4 in mouse MB49 cells transduced with lentivirus encoding scramble shRNA (shControl) and shRNAs for TLR4 (sh1TLR4 and sh2TLR4). Results are means ± SEM of measurements from three separate experiments. *$P < 0.05$ (Student's t-test).

Supplementary Figure 2. BCG evokes intracellular Ca^{2+} signaling in bladder cancer cells. Human RT4 cells (**A**) and mouse MB49 cells (**B**) exposed to BCG ($4x10^6$–$6x10^7$ cfu/mL) exhibit Ca^{2+} signaling. Single-cell traces represent typical responses.

Supplementary Figure 3. Scrutinizing the BCG-induced Ca^{2+} signal cascade in bladder cancer cells. Ca^{2+} signaling in T24 bladder cancer cells exposed to BCG ($4x10^6$–$6x10^7$ cfu/mL) together with the SERCA pump inhibitor cyclopiazonic acid (CPA) (**A**), $InsP_3R$ inhibitors 2-aminoethoxydiphenylborane (2APB) (**B**) or Xestospongin D (**C**), PLC-inhibitor U73122 (**D**) or its negative control U73343 (**E**), siRNAs against PLCβ3 (**F**) or PLCγ (**G**), G-protein couple receptor inhibitor pertussis toxin (PTX) (**H**), or phosphatidylinositol 3-kinase inhibitor Wortmannin (**I**). Single-cell traces represent typical responses.

Supplementary Figure 4. Screening substances in BCG. Polyacrylamide gel electrophoresis of the BCG mixture. Weight markers are indicated in kDa.

Supplementary movie legends

Supplementary Movie 1. BCG-induced Ca^{2+} signaling in bladder cancer cells. Human T24 bladder cancer cells loaded with the Ca^{2+}-probe Fluo-3/AM and exposed to BCG at time point 300 sec. Time-lapse recordings were performed with sampling frequency 0.2 Hz.

Ibarra and Karlsson et al. - Supplementary Figure 1

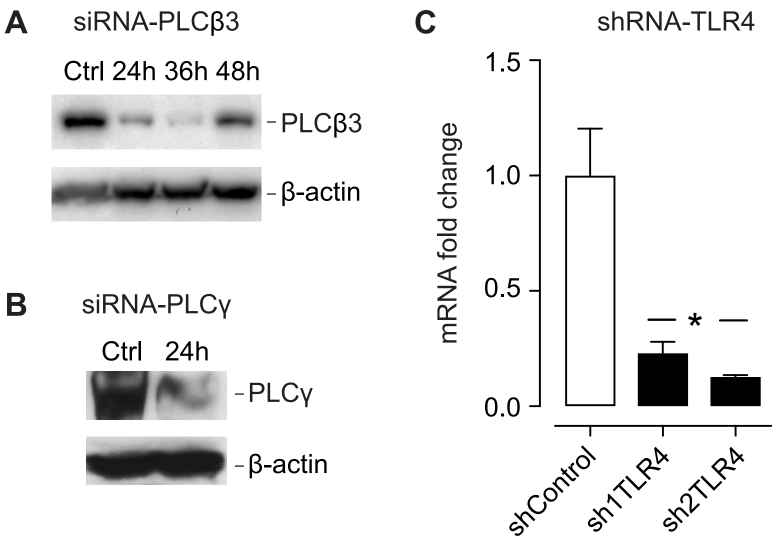

Ibarra and Karlsson et al. - Supplementary Figure 2

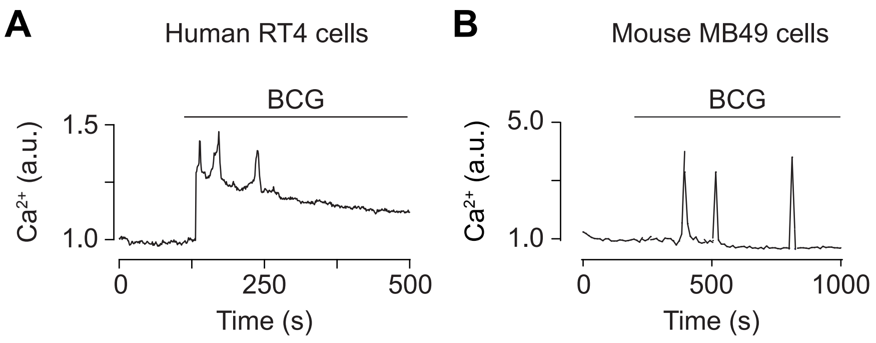

Ibarra and Karlsson et al. - Supplementary Figure 3

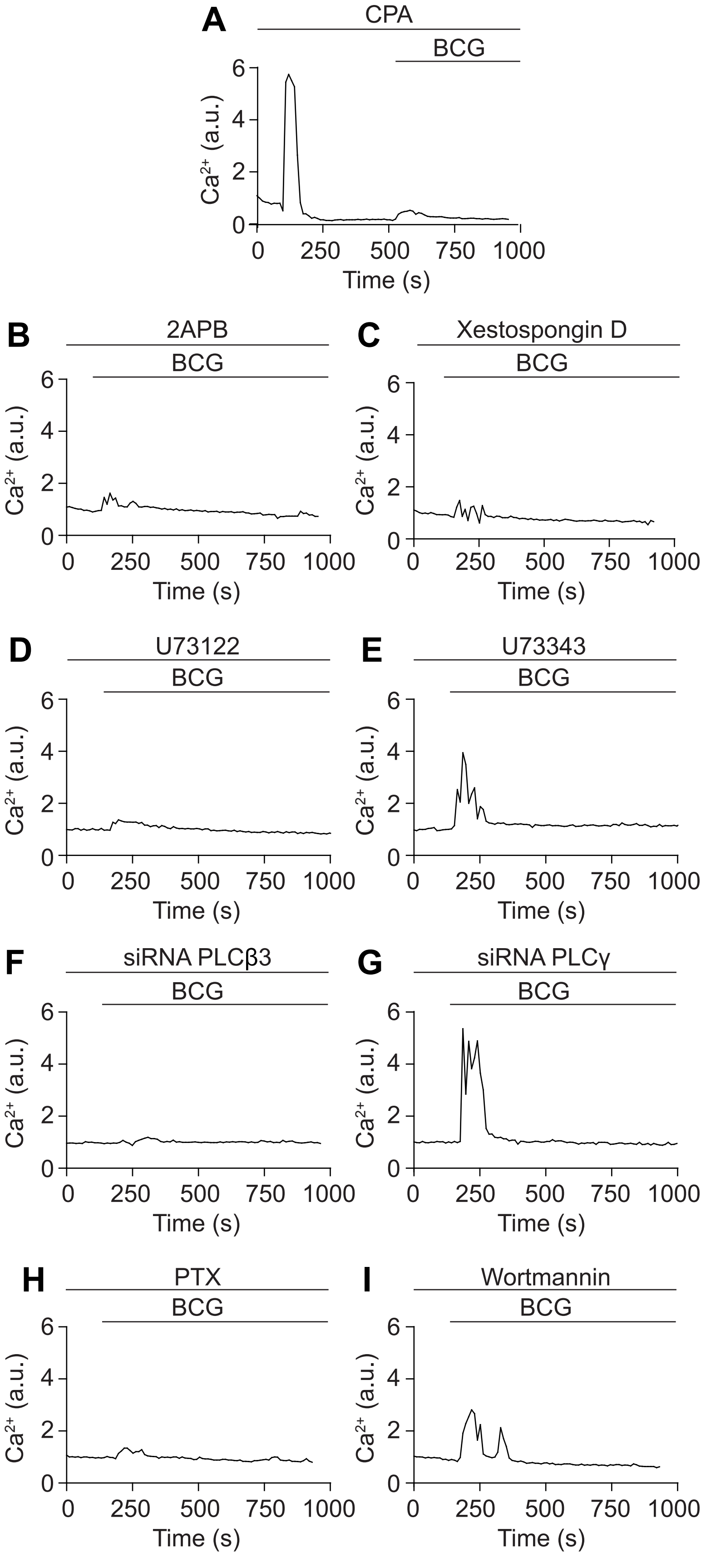

Ibarra and Karlsson et al. - Supplementary Figure 4

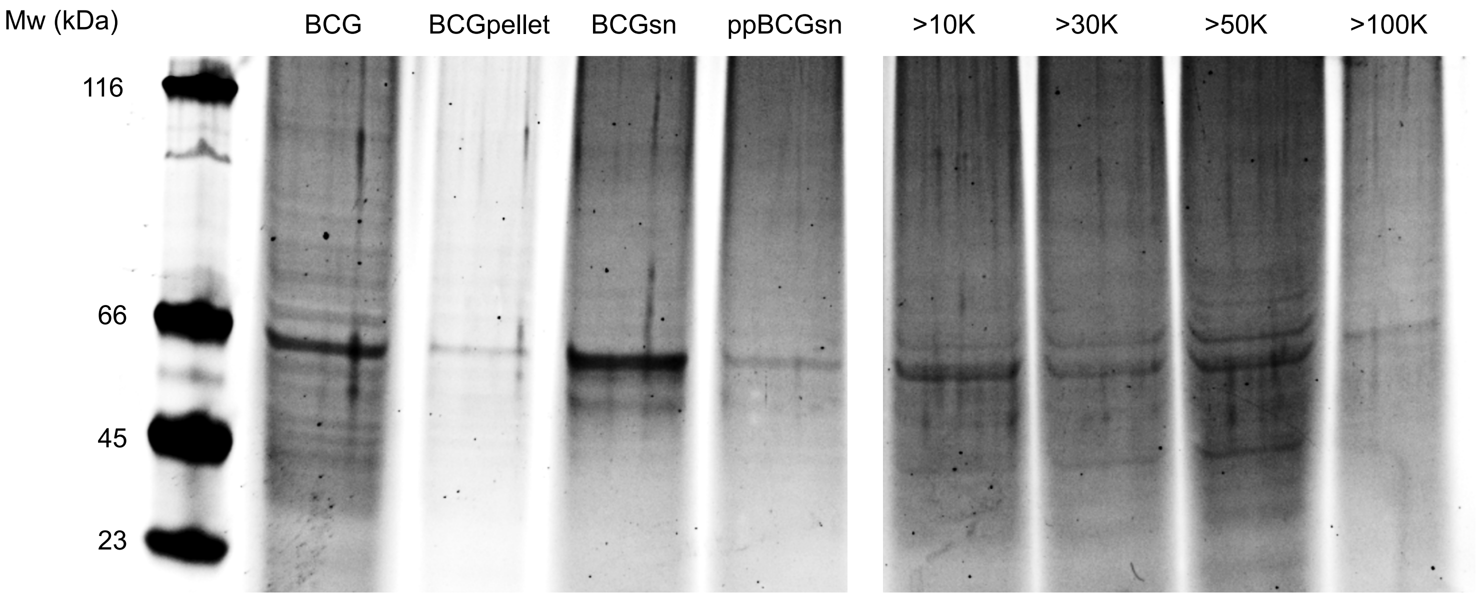

Supplement: Supplementary file 1 — Fig. S1. Validating knock‐down efficiency of siRNA and shRNA. Fig. S2. BCG evokes intracellular Ca2 + signaling in bladder cancer cells. Fig. S3. Scrutinizing the BCG‐induced Ca2 + signal cascade in bladder cancer cells. Fig. S4. Screening substances in BCG. Polyacrylamide gel electrophoresis of the BCG mixture. [file MOL2-13-202-s001.pdf]
